# α-Pinene: Docking Study, Cytotoxicity, Mechanism of Action, and Anti-Biofilm Effect against *Candida albicans*

**DOI:** 10.3390/antibiotics12030480

**Published:** 2023-02-28

**Authors:** Daniela Bomfim de Barros, Luanna de Oliveira e Lima, Larissa Alves da Silva, Mariana Cavalcante Fonseca, Rafael Carlos Ferreira, Hermes Diniz Neto, Danielle da Nóbrega Alves, Walicyranison Plinio da Silva Rocha, Luciana Scotti, Edeltrudes de Oliveira Lima, Marianna Vieira Sobral, Lúcio Roberto Cançado Castellano, Juliana Moura-Mendes, Felipe Queiroga Sarmento Guerra, Márcia Vanusa da Silva

**Affiliations:** 1 Department of Biochemistry, Federal University of Pernambuco, Recife 50670-901, PE, Brazil; 2 Department of Pharmaceutical Sciences, Paraíba Federal University, João Pessoa 58051-900, PB, Brazil; 3 Human Immunology Research and Education Group-GEPIH, Technical School of Health, Federal University of Paraíba, João Pessoa 58051-900, PB, Brazil; 4 Postgraduate Program in Natural Products and Synthetic Bioactive, Federal University of Paraíba, João Pessoa 58051-900, PB, Brazil; 5 University Hospital Julio Muller, Federal University of Mato Grosso, Cuiabá 78060-900, MT, Brazil; 6 Department of Clinical and Social Dentistry, Federal University of Paraíba, João Pessoa 58051-900, PB, Brazil; 7 Cheminformatics Laboratory, Postgraduate Program in Natural Products and Synthetic Bioactive, Quality Management, University Hospital, Federal University of Paraíba, João Pessoa 58051-900, PB, Brazil; 8 Centro Multidisciplinario de Investigaciones Tecnológicas, Universidad Nacional de Asunción, San Lorenzo 111421, Paraguay

**Keywords:** natural product, terpenes, fungicidal activity, biofilm, candidiasis, in silico, in vitro

## Abstract

*Candida albicans* is associated with serious infections in immunocompromised patients. Terpenes are natural-product derivatives, widely studied as antifungal alternatives. In a previous study reported by our group, the antifungal activity of α-pinene against *C. albicans* was verified; α-pinene presented an MIC between 128–512 µg/mL. In this study, we evaluate time-kill, a mechanism of action using in silico and in vitro tests, anti-biofilm activity against the *Candida albicans,* and toxicity against human cells (HaCaT). Results from the molecular-docking simulation demonstrated that thymidylate synthase (−52 kcal mol^−1^), and δ-14-sterol reductase (−44 kcal mol^−1^) presented the best interactions. Our in vitro results suggest that α-pinene’s antifungal activity involves binding to ergosterol in the cellular membrane. In the time-kill assay, the antifungal activity was not time-dependent, and also inhibited biofilm formation, while rupturing up to 88% of existing biofilm. It was non-cytotoxic to human keratinocytes. Our study supports α-pinene as a candidate to treat fungal infections caused by *C. albicans.*

## 1. Introduction

The advance of medicine has increased life expectancy and aided in the treatment of many diseases, yet this has also caused much greater susceptibility to opportunistic fungal diseases [1]. Patients hospitalized and receiving chemotherapy, bone marrow transplants, and those with acquired immunodeficiency syndrome (AIDS) and other immune-deficiency diseases are often exposed to invasive fungal infections (IFI) [2]. IFIs are frequently associated with *Aspergillus*, *Cryptococcus,* and *Candida* [1]. *Candida* species are responsible for many cases of septicemia, bringing high mortality rates and medical costs as well [3,4]. *Candida albicans*, a fungus that inhabits the gastrointestinal and genitourinary tracts, and the oral and conjunctive microbiota is the species most isolated as an etiological agent of candidiasis [2,5]. *Candida albicans* is the third most-commonly isolated microorganism in bloodstream infections in hospitalized patients [6]. The World Health Organization (WHO) recently published a list of priority pathogenic fungi that pose a threat to public health. Of the 19 fungal species mentioned in the document, *Candida albicans* was noted as a high-priority- (critical) level pathogen, due to the risks of morbidity and mortality. The publication is intended to strengthen the global response to fungal infections and use of antifungal agents [7].

Common antifungals used in *Candida* treatment, the azoles, are a first choice for their wide activity, low cost, and for being safe [8,9]. Polyenes, and in more severe cases of candidemia, fluoropyrimidines, and echinocandins are also prescribed, the latter being the best option for patients with neutropenia [8,10,11]. However, treatment is still a challenge [9], as it can lead to cytotoxic effects and antifungal resistance [10], often caused by self-diagnosis and indiscriminate use. The high relapse rate has created a rather worrying scenario [2,8]. Antifungal-therapy failures result in prolonged illness, higher toxicity, and more deaths [12]. To reverse this situation, the WHO is attempting incentivize more research in the area [7]. 

In view of the known increasing resistance to synthetic antifungals and to their toxicity, it has become more urgent to promote studies involving natural substances [2]. Natural products and their derivatives have long been a source of bioactive compounds, including those with antifungal activity, low toxicity, and low costs [13,14]. Synthetic substances are expensive to synthesize, and new synthetic compounds that overcome pre-clinical and clinical stages of fungal infections are rare [15]. Among the more promising natural compounds are the monoterpenes, such as pinenes. These can be found in the essential oils of coniferous trees (pine), and in turpentine, rosemary, and lavender [13]. One of the most relevant pinenes is α-pinene, which presents antifungal activity against *C. albicans*, *C. parapsilosis*, and *C. tropicalis* [16,17,18].

As reported by our group [19], the antifungal activity of α-pinene against planktonic strains of *C. albicans* has been verified, whether alone or in association with other drugs. A-pinene presented an MIC between 128–512 µg/mL and the MFC presented the same values, which demonstrated its fungicidal effect. Despite the promising results of the study, better assessment of the effects of α-pinene against *C. albicans*, such as biofilm activity, and possible mechanism action, as well as cytotoxicity, are needed.

The present work aimed to perform molecular-docking, toxicity, and mechanism-of-action analysis, anti-biofilm-effect evaluations, and time-kill assays for α-pinene against *C. albicans*.

## 2. Results

### 2.1. Docking Prediction

As demonstrated in Table 1, α-pinene presented negative ligand–receptor interaction energies for the molecular targets, demonstrating affinities mainly for thymidylate synthase (−52 kcal mol^−1^) and δ -14-sterol reductase (−44 kcal mol^−1^).

**Figure 1:**
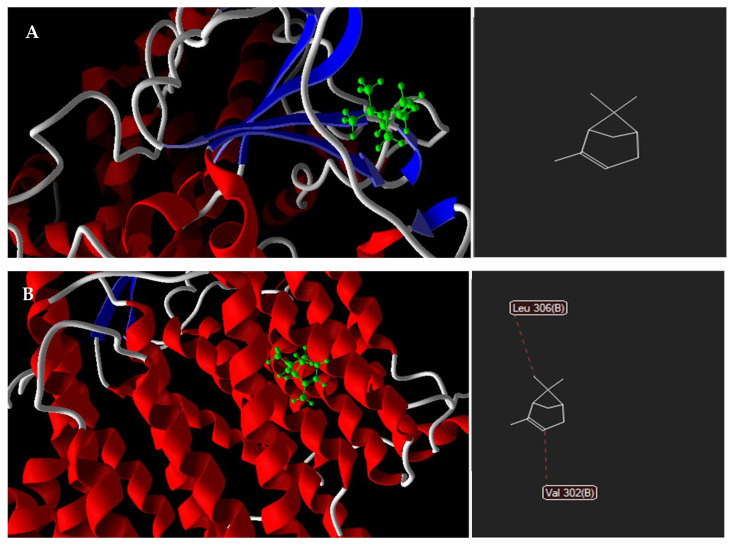

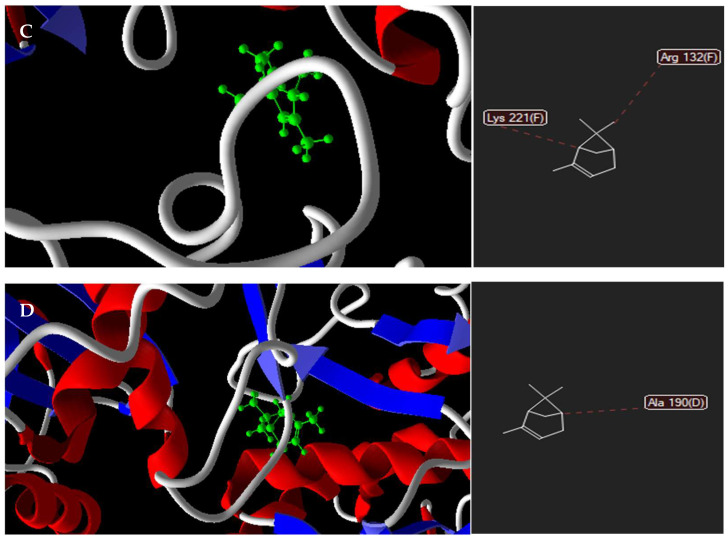
PDB ID protein—α-pinene interaction maps. (**A**) Map of interactions between the PDB protein ID 3JUV (14-α-demethylase) and α-pinene; (**B**) Map of interactions between the PDB ID 4QUV protein (δ -14-sterol reductase) and α-pinene; (**C**) Map of interactions between the PDB ID 2J0Y protein (1,3-β-glucan synthase) and α-pinene; (**D**) Map of interactions between the PDB ID 3QJ7 protein (thymidylate synthase) and α-pinene.

Figure 1 shows that the α-pinene interactions with the amino-acid residues are mediated by hydrogen bonds (depicted in blue). The structures (in blue, red, and green, respectively), correspond to hydrogen, steric, and electrostatic bonds.

### 2.2. Sorbitol and Exogenous-Ergosterol Assays

Sorbitol is an osmotic protector that minimizes the effects of chemical agent(s) on the fungal cell wall. In our study, the presence of an osmotic protector did not affect the minimum concentration of α-pinene required to inhibit cell growth for *C. albicans* ATCC 76485, suggesting that its mechanism of action is not related to disruption of the cell wall (Table 2).

On the other hand, our findings showed that the MIC of α-pinene increased (from 128 µg/mL to >512 µg/mL) in the presence of exogenous ergosterol. This suggests the disruption of cell-membrane permeability is likely related to membrane-ergosterol binding (Table 2).

### 2.3. Time-Kill Assay

**Figure 2:**
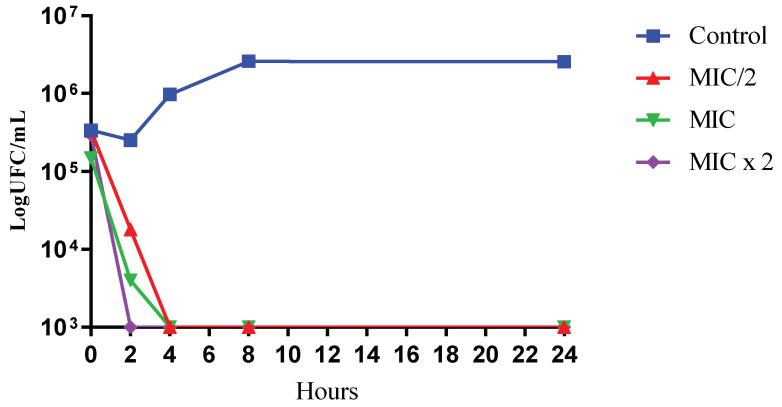
Time-kill curve for *C. albicans* ATCC 76485 when exposed to various concentrations of α-pinene at different time intervals. MIC: Minimum Inhibitory Concentration CFU: Colony Forming Unit.

While subjected to different concentrations of the α-pinene, yeast growth was analyzed over time. *C. albicans* ATCC 76485 was subjected to the experimental method for microbial death kinetics (Figure 2). The graph presents the log10 of CFU/mL versus time of exposure in the presence of α-pinene (MIC/2, MIC, and MIC × 2), the standard antifungal, and the control. The graph reveals that the α-pinene concentrations of MIC/2 and MIC present fungicide activity at 4 h of exposure, since there was a reduction of less than 3 log_10_ CFU/mL of the initial inoculum. This behavior was also seen for the MIC × 2 concentrations at up to 2 h of exposure. Therefore, it was possible to identify in the time-kill tests the fact that fungicidal activity of α-pinene is not time-dependent.

### 2.4. α-Pinene Anti-Biofilm Activity

**Figure 3:**
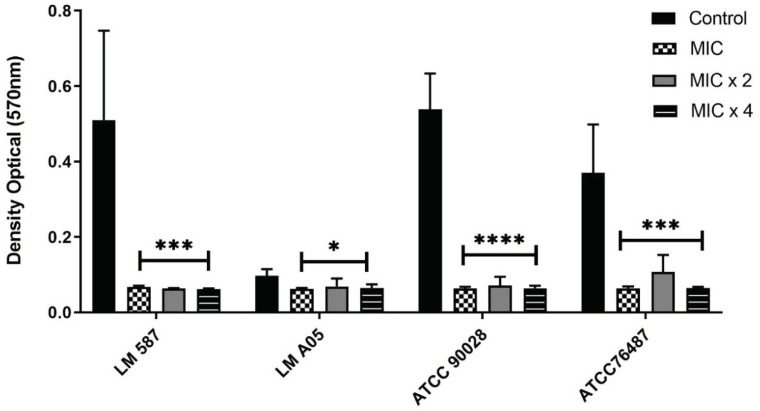
Rupture of mature *C. albicans* biofilm after exposure to different concentrations of α-pinene. * *p* < 0.05; *** *p* < 0.001; **** *p* < 0.0001 as compared to the control group. MIC: Minimum Inhibitory Concentration; LM: from the UFPB Mycology Laboratory.

Rupture percentages of existing (mature) biofilm are shown in Figure 3. According to the data obtained, we observed that α-pinene presented significant rates of biofilm rupture at all tested concentrations (MIC, MIC × 2, and MIC × 4) and for all strains (except the A05 strain, which was less than 50%). The highest rupture rates (between 71% and 88%) were observed for the higher concentrations of the substance, and there were no significant differences between biofilm rupture rates between species when tested at the same concentrations.

### 2.5. Cytotoxicity Assay against HaCaT Cells

**Figure 4:**
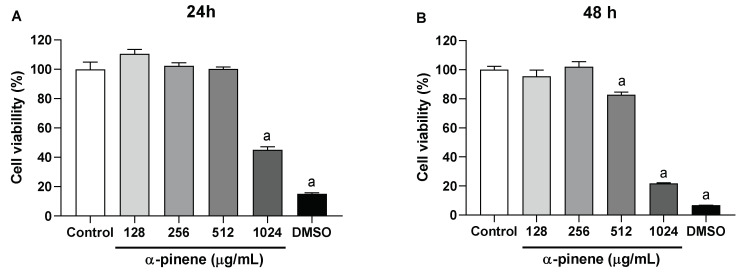
Cytotoxicity of α-pinene against human cell line HaCaT at 24 and 48 h. (**A**): Cell viability after 24 h of α-pinene treatment. (**B**): Cell viability after 48 h of α-pinene treatment. Data were analyzed with GraphPad Prism 8.0 software and presented as the mean ± SEM of three independent tests performed in quadruplicate. Significant difference (*p* < 0.05) when compared to drug-free growth control (a).

After 24 h of treatment, α-pinene induced significant cytotoxicity at 1024 µg/mL, with 54.98 ± 2.22% inhibition of cell proliferation. At 48 h of incubation, significant α-pinene cytotoxic effect was observed between 512 and 1024 µg/mL, with the respective inhibition of cell-growth percentages of 17.29 ± 1.93% and 78.11 ± 0.32% (Figure 4). 

## 3. Discussion

In silico assays, particularly molecular docking, are important tools in medicinal chemistry. They serve to predict molecular interactions between structures, generally a receptor protein and a ligand. Docking analysis calculates the energy values needed to perform the coupling in each of the possible linking sites [20]. There are no previous studies reporting the interaction of α-pinene with the tested enzymes. In the study, the α-pinene-thymidylate synthase interaction presented the lowest binding energy, leading to a prediction that the phytoconstituent has the ability to bind to this receptor with consequent activity involving fungal-DNA synthesis [21].

Many drugs available for clinical use interact directly with ergosterol, causing damage to the fungal cell membrane. It can be verified whether test products interact directly with ergosterol, and if the effects of the selected compounds on the fungal cell are due to binding to ergosterol present in the membrane, since in the presence of exogenous ergosterol in the culture medium, prevention of membrane-ergosterol binding occurs. The MIC of the product tends to increase in the presence of exogenous ergosterol, because it will need higher concentrations to interact with membrane ergosterol [22]. The exogenous-ergosterol and sorbitol assays in the present work suggest that the antifungal activity of α-pinene may be related to ergosterol binding in the fungal cytoplasmic membrane, and the data described is unprecedented.

Yeast growth over time was analyzed using various concentrations of test product and including a viable cell count, verifying fungistatic or fungicidal action, and the analysis of the microorganism–test-product interaction, to characterize concentration/activity dynamics over time [23].

It was seen that α-pinene presents a fungicidal effect, and that this effect occurs after 4 h of exposure to the drug, corroborating what was observed in our previous study [19], which demonstrated a fungicidal effect through the determination of MIC and MFC.

Silva et al. [13], aiming to determine the death-time curve of *C. albicans* and MRSA, observed that α-pinene positive enantiomers were fungicidal after 60 min of exposure. Bactericidal effect was observed after 6 h of incubation. Discordant with our study, this result can be explained by the fact that a different strain of *C. albicans* was used. Saracino et al. [24], observed no α-pinene effect against *C. albicans* in the first 6 h, a decrease in cell viability at 8 h, and complete cell death at 24 h. 

Biofilms are sessile microbial communities surrounded by self-produced extracellular matrices which are considerably more resistant to conventional antifungals and require higher effective concentrations. Assaying anti-biofilms is therefore important [25,26]. The data obtained demonstrate that α-pinene was able to perform mature biofilm disruption at all concentrations tested (MIC, MIC × 2, and MIC × 4). 

The α-pinene biofilm-formation-inhibition assay has already been conducted, demonstrating 100% inhibition at the MIC [13]. However this is the first study that explores the potential of α-pinene in the eradication of preexisting biofilm. HaCaT cells, an immortalized keratinocyte lineage, have been widely used in in vitro assays, and are an important tool for studies of epidermal homeostasis and its pathophysiology [27]. In this study, the cytotoxicity of α-pinene against HaCaT cells at 24 h and 48 h was also evaluated. At 24 h, the test substance caused cell death only at the maximum concentration, 1024 μg/mL. At 48 h, the result was similar, with viability above 50% MIC, being cytotoxic only at the highest concentration.

Similar results were also obtained in a study of α-pinene photo-protective activity. HaCaT cells were subjected to a single UVA-radiation dose in the presence of the substance (30 µM), and different cellular endpoints were analyzed. The 31.25 µM concentration revealed a viability percentage above 50%, with α-pinene being cytotoxic only at 1000 µM. The study demonstrated that α-pinene prevents UVA-induced oxidative stress, and inhibits ROS generation, and lipid peroxidation in HaCaT cells [28].

Karthikeyan et al. [29], also demonstrated that the substance prevents UVA-induced photo-aging through the maintenance of intracellular antioxidants, modulation of Bax/Bcl-2 expression, and preventing cell death by hindering the activation of apoptotic-effect mediators, such as the expression of caspase-3. The monoterpene also suppressed metalloproteinases and inhibited the UVA-induced activation of proangiogenic and inflammatory-protein expression, preventing the photo-aging process.

In the docking tests, it was observed that α-pinene presents better interaction with thymidylate synthase, revealing fungicidal activity against *Candida albicans* through a mechanism of action likely related to ergosterol complexation. In the time-kill assay, it was demonstrated that the fungicidal activity of α-pinene is not time dependent, and that it also inhibits biofilm formation, with up to 88% rupture of preexisting biofilms. The cytotoxicity test revealed that α-pinene was not toxic to human keratinocytes.

## 4. Materials and Methods

### 4.1. Test Substance, Antifungal Drugs, and Culture Media

The test substance α-pinene, the antifungals (amphotericin B; caspofungin), ergosterol, and dimethylsulfoxide (DMSO) were commercially obtained from the manufacturer Sigma Aldrich^®^. Sorbitol was obtained from VETEC Química Fina Ltd.a-Rio de Janeiro/RJ. The culture medium Sabouraud Dextrose Agar (SDA) (Difco Laboratories, Detroit, MI, USA) was used for the maintenance of the *Candida* strains. The liquid culture medium Roswell Park Memorial Institute (RPMI)-1640-L-glutamine (without sodium bicarbonate) (Sigma-Aldrich^®^, São Paulo, SP, Brazil) was used for the antifungal assays. Both culture media were prepared according to the manufacturer’s instructions. The α-pinene was dis-solved in DMSO (with a final concentration of 0.13% and 1% for the cytotoxicity Aasay and anti-fungal activity, respectively).

### 4.2. Microorganisms

The following strains were used in the experiments: *Candida albicans* ATCC 76485, *C. albicans* ATCC-90028, *C. albicans* LM 587, and *C. albicans* A05. The strains were provided by the Mycology Laboratory at the Federal University of Paraíba-UFPB. The microorganisms were kept in SDA at 4 °C until the realization of the tests. Prior to each experiment, the cells were reactivated on SDA agar plates for inoculum preparation, and the microorganism colonies were suspended in a 0.85% sterile NaCl solution, and then adjusted to the 0.5 scale of McFarland standard [30,31]. 

### 4.3. Molecular Docking 

The structure of α-pinene was downloaded from PubChem (https://pubchem.ncbi.nlm.nih.gov/; accessed on 1 February 2022), in sdf format. The receptors investigated were: 14-α-demethylase, δ-14-sterol reductase, 1,3-β-glucan synthase, and thymidylate synthase. These were downloaded from the Protein Data Bank (http://www.rcsb.org/pdb/home/home.do accessed on 5 February 2022) with the respective IDs: 3UV [32], 4QUV [33], 2J0Y [34], and 3QJ7 [35]. The ligand and selected receptors were docked using the Molegro Virtual Docker, v. 6.0.1 (MVD) [36].

The proteins and ligand structures were prepared using the default parameter settings in the software. Characteristic models that were expected to be relevant for binding the α-pinene ligand were also generated. The MolDock evaluation model [GRID] was used as the evaluation function, and the MolDock search facility was used [37].

### 4.4. Mechanism of Action

#### 4.4.1. Effect on the Fungal Cell Wall

To evaluate the fungal-cell-wall–terpene interaction, the MICs of the products were evaluated in the presence and absence of an osmotic stabilizer, 0.8 M sorbitol. If the product acted on the fungal cell wall, it would cause cell lysis in the absence of the osmotic stabilizer, but would allow fungal growth in the presence of sorbitol. Thus, the antifungal products´ MIC’s were compared in the absence and presence of 0.8 M sorbitol. 

Determination of the product MICs (in the presence of sorbitol) was performed similarly to the normal MIC determinations. In each plate well, 100 µL of RPMI 1640 liquid medium previously supplemented with sorbitol (MW = 182.17) (VETEC Química Fina Ltd.a—Rio de Janeiro/RJ) was added, both doubly concentrated. Subsequently, 100 µL of the product solution, also doubly concentrated, was added to the wells of the first row of the plate. Through a serial dilution (ratio of two), concentrations of 1024 µg/mL to 0.125 µg/mL of the products were obtained. In the case of sorbitol, each well presented a final concentration of 0.8 M. Finally, 10 µL of the fungal inoculum was added to the wells, and each column of the plate referred to a specific fungal strain.

A microorganism control was performed, in which 100 µL of the same RPMI 1640, sorbitol (0.8 M), and 10 µL of the inoculum of each species were added to the well. A sterility control was also performed, placing 200 µL of RPMI 1640 in a well without the fungal suspension. And finally, a control with DMSO (DMSO 1% + inoculum + RPMI 1640) was performed.

The same procedure was also performed with the antifungal caspofungin serving as a control, since it acts against the growth of fungi by inhibiting β-1,3-D-glucansynthase present in the cell wall. The plates were incubated for 24 h at a temperature of 35–37 °C, and a subsequent visual reading of the plates was performed [22,38].

#### 4.4.2. Ergosterol Binding Assay

Determination of the product MICs with and without exogenous ergosterol (400 µg/mL) was performed in the RPMI 1640 medium, according to the methodology described above. The culture medium (RPMI 1640) was used in the absence and presence of ergosterol (Sigma-Aldrich^®^, St. Louis, MO, USA). Finally, a control was performed with amphotericin B, which has a known mechanism of action of interaction with membrane ergosterol [39].

### 4.5. Time-Kill

The microorganism death-time curve was used to assess the minimum time required for the death of the fungal strains in the presence of the phytoconstituent (26). The yeast strains´ response was observed for minimum inhibitory concentrations of the product during 24 h. First, 1 mL of the fungal suspension was inoculated into 9 mL of SDA with the substance in increasing concentrations, with MIC/2, MIC and MIC × 2, respectively, corresponding to 128 µg/mL, 256 µg/mL and 512 µg/mL. Fungal-growth control was also performed. At intervals of 0 h, 2 h, 4 h, 8 h, and 24 h after incubation, 10 µL of the inoculum was uniformly seeded on SDA plate. The plates were incubated at 35 ± 2 °C for 24–48 h. At the end of the incubation period, the existing CFU in the Petri dishes were counted, and the numbers per mL of solution in each period were determined for each substance in its 3 concentrations. The experiment was performed in duplicate, and the time-to-death curves were made by plotting the mean colony count (log10 CFU/mL) as a function of time (in hours) with the GraphPad Prism software. Antifungal activity for the substance was considered when there was growth greater than or equal to 3 log10 (99.9%), and fungistatic activity when there was a reduction in growth of less than 3 log10 (<99.9%) CFU/mL [40].

### 4.6. Biofilm Eradication

In order to observe whether the monoterpene was capable of causing damage to mature biofilm, 10 µL of *Candida albicans* fungal inoculum, strains LM 587, LM A05, ATCC 90028, and ATCC 76485 were incubated for 48 h at 35 ± 2 °C in 100 µL of RPMI broth, to form biofilms. After discarding the contents of the wells, 100µL of RPMI 1640 broth, containing different concentrations of α-pinene (MIC, MIC × 2, and MIC × 4) was added and incubated again for 48 h at 35 ± 2 °C. At the end of the incubation period, quantification was performed using the crystal-violet assay. Briefly, the biofilm-coated wells of the 96-well plates were washed twice with 150 µL of PBS, and then air-dried for 45 min. Afterwards, each well was stained with 110 µL of 0.4% aqueous crystal violet, also for 45 min. Then, each well was washed four times with 350 µL of sterile distilled water and immediately stained with 200 µL of 95% ethanol. Finally, after another 45 min of decolorization, 100 µL of the solution was transferred to a new well and the amount of crystal-violet stain in the decolorizing solution was measured with a microtiter plate reader (SpectraMAX 340 Adjustable Microplate Reader; Molecular Devices Ltd., Sunnyvale, CA, USA) at 595 nm. The analyses were performed in triplicate, and the results expressed as the arithmetic mean of the absorption values obtained, these being plotted in graphs with the statistical software GraphPad Prism GraphPad Prism version 8.0.0 for Windows, GraphPad Software, San Diego, CA, USA. To calculate the percentage of rupture of the biofilm formed, the formula used was: biofilm rupture % = 100 − [(ABS590 test/ABS590 control) × 100] [41]. 

### 4.7. Cytotoxicity Assay against HaCaT Cells

To evaluate the cytotoxicity of α-pinene, an MTT assay was performed. The human cell line HaCaT (keratinocytes) were cultured in DMEM medium supplemented with 10% fetal bovine serum, 100 U/mL penicillin, and 100 μg/mL streptomycin at 37 °C in a humidified atmosphere with 5% CO_2_. Cells were seeded into 96-well plates at a density of 3 × 10^5^ cell/mL. Following a 24 h period, the cells were incubated, together with α-pinene (128, 256, 512, or 1024 µg/mL) dissolved in DMSO (0.13%). Each experiment was performed in quadruplicate. The positive control was treated with DMSO (20%). Finally, a control with DMSO (DMSO+ cell line HaCaT + DMEM) was performed. After incubation for 24 h and 48 h, the supernatant was discarded, and 3-(4,5-dimethylthiazol-2-yl)-2,5-diphenyltetrazolium bromide (MTT) solution (5 mg/mL) was added, and these were incubated together for another 4 h. The formazan deposited was dissolved with Sodium Dodecyl Sulfate (SDS) (100 μL). The optical densities were measured using a microplate reader (Synergy HT, BioTek^®^, Santa Clara, CA, USA) at 570 nm [42].

## 5. Conclusions

The α-pinene presented fungicidal activity against *Candida albicans* through a mechanism of action likely related to ergosterol complexation, yet other pathways may be related. The docking test showed that the best molecular interaction for α-pinene was with thymidylate synthase. In the time-kill assay, its antifungal activity was not time-dependent, and α-pinene also inhibited biofilm formation, while rupturing up to 88% of existing or preformed biofilm. α-pinene was non-cytotoxic to human keratinocytes. Our study supports α-pinene as a candidate to treat fungal infections caused by *C. albicans.*

## Figures and Tables

**Table 1:** Binding-energy (kcal mol^−1^) values of α-pinene with the molecular targets 1,3-β-glucan synthase (2JOY), δ-14-sterol reductase (4QUV), 14-α-demethylase (3JUV), and thymidylate synthase (3QJ7).

	Compounds			
	14-α-Demethylase (3JUV)	δ-14-Sterol Reductase (4QUV)	1,3-β-Glucan Synthase (2J0Y)	Thimidylate Synthase (3QJ7)
Energy (kcal/mol)	−16	−44	−32	−52

**Table 2:** MIC values (μg/mL) of drugs in the absence and presence of sorbitol (0.8 M) and exogenous ergosterol (1.008 mM) against *C. albicans* ATCC 76485. Values are expressed in μg/mL.

Drugs	Sorbitol		Ergosterol	
	Absence	Presence	Absence	Presence
α-pinene	128	128	128	>512
Caspofungin	0.062	2	NA	NA
Amphotericin B	NA	NA	1.25	>512

NA: not applicable.

## Data Availability

Not applicable.
